# New Approach in the Application of Conjugated Polymers: The Light-Activated Source of Versatile Singlet Oxygen Molecule

**DOI:** 10.3390/ma14051098

**Published:** 2021-02-26

**Authors:** Agata Blacha-Grzechnik

**Affiliations:** Faculty of Chemistry, Silesian University of Technology, Strzody 9, 44-100 Gliwice, Poland; agata.blacha@polsl.pl; Tel.: +48-322371024; Fax: +48-322371509

**Keywords:** conjugated polymers, conductive polymers, singlet oxygen, reactive oxygen species, photosensitizers, PDT, PACT

## Abstract

For many years, the research on conjugated polymers (CPs) has been mainly focused on their application in organic electronics. Recent works, however, show that due to the unique optical and photophysical properties of CPs, such as high absorption in UV–Vis or even near-infrared (NIR) region and efficient intra-/intermolecular energy transfer, which can be relatively easily optimized, CPs can be considered as an effective light-activated source of versatile and highly reactive singlet oxygen for medical or catalytic use. The aim of this short review is to present the novel possibilities that lie dormant in those exceptional polymers with the extended system of π-conjugated bonds.

## 1. Introduction

Singlet oxygen, ^1^O_2_, is one of the Reactive Oxygen Species (ROS), that has been under high research interest for more than 50 years now [1]. Molecular oxygen possesses two singlet excited states (a^1^Δ_g_ and b^1^Σ_g_+) that, unlike ground state triplet oxygen, do not have unpaired electrons on the π-antibonding orbital [1,2]. The b^1^Σ_g_+ form is highly unstable and rapidly decays to a^1^Δ_g_. The latter, commonly named as singlet oxygen, is a relatively long-lived (45 min in gas phase and up to 10^−3^ s in solution) species, because the transition to ground triplet state, X^3^Σ_g_^−^, is spin-forbidden. Singlet oxygen has stronger oxidizing properties than triplet state molecular oxygen, which results in its higher reactivity and electrophilic character. Hence, it has become an interesting candidate for the application in various areas of medicine, chemistry or environmental science [1,2].

**Figure 1:**
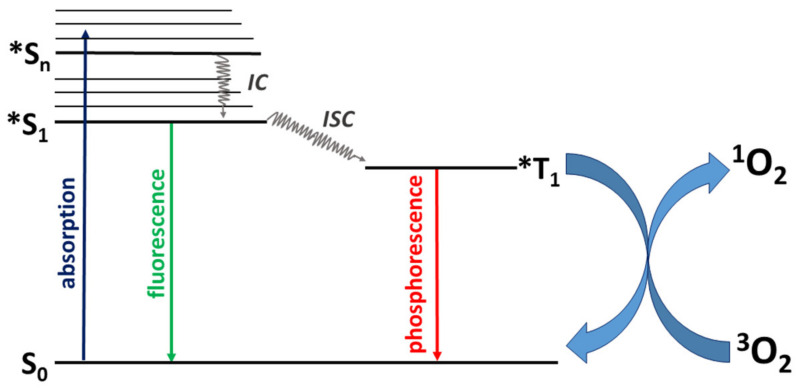
Jablonski diagram of the photosensitization process. S_0_–singlet ground state of photosensitizer, *S_1,n_–singlet excited states of photosensitizers, *T_1_–triplet excited state of photosensitizer, *IC*–internal conversion, and *ISC*–intersystem crossing.

Among several methods of singlet oxygen formation, chemical and photochemical routes appear as the most prominent. The first one may consist of the decomposition of organic or inorganic oxygen-bearing species [3,4,5]. Even though attention nowadays is paid to development of the chemical methods, the photochemical approach for singlet oxygen generation still remains the most commonly applied. ^1^O_2_, together with other forms of ROS, can be produced in the so-called photosensitization process, where the appropriate photoactive molecule, i.e., photosensitizer (PS), is excited from its ground state, S_0_, into singlet excited state, S_n_, by light illumination. In the next steps, the relaxation (internal conversion, *IC*) of S_n_ gives the sensitizer’s lowest singlet excited state, and further, the triplet state with longer lifetime, T_1_, via the intersystem crossing (*ISC*). The photosensitizer being in the triplet state can further transfer its energy to ground state triplet oxygen to form a singlet oxygen molecule (see Jablonski Diagram, Figure 1) [1,2]. The excited photosensitizer can also react with other substrates by the electron transfer or hydrogen abstraction, to form radicals or radical ions that can subsequently react with triplet oxygen to form other ROS, such as the superoxide anion [1,2,6].

Several review papers discussing the details of the chemical and physical properties of singlet oxygen, its generation, detection and application, have been published until now [1,2,6,7,8,9]. Hence, these issues will not be discussed further in this short review. This work aims to show new trends in the application of conjugated polymers (CPs) as efficient organic photosensitizers.

There are several requirements that should be fulfilled by a molecule to be considered as a good photosensitizer. First of all, it should possess a high absorption coefficient at the wavelength consistent with the illumination source. Second of all, the triplet state should be characterized by an energy higher than 95 kJ/mol and long lifetime (more than 1 µs) to allow for the transfer of energy to triplet oxygen. Additionally, the triplet state of PS should be formed with a high quantum yield from the singlet state. Finally, photosensitizers should possess high photostability [1,10]. Moreover, for the medical applications PS must have low dark toxicity [11]. Among the classical photosensitizers, the following groups can be defined: (a) organic dyes and aromatic hydrocarbons, (e.g., rose bengal, methylene blue, anthracene), (b) porphyrins, phthalocyanines, (c) transition metals (e.g., ruthenium, platinum, palladium) complexes, and (d) inorganic semiconductors (e.g., TiO_2_, ZnO) [1,12,13]. They are very efficient in the photogeneration of singlet oxygen; however each of them possesses some weaknesses. For example, dyes are usually monochromophore molecules that have only one, usually narrow absorption band in the visible region, which in turn results in rather noneffective usage of the solar light. Another critical issue is the efficiency of the intersystem crossing process. Hence, the research for the perfect photosensitizer is still underway. It has been shown that the visible-light absorption can be significantly enhanced by the attachment of various light-harvesting antennas ([14,15]), while *ICS* can be facilitated by the introduction of a heavy atom or by lowering of the energy difference between the S_1_ and T_1_ states. These approaches are accomplished in the novel triplet organic photosensitizer [16]. Moreover, other groups of compounds have been also proposed in recent years as efficient sources of singlet oxygen—e.g., pristine or functionalized carbon nanostructures (fullerenes, nanotubes, graphene oxide, graphene quantum dots) [17,18,19,20,21,22], or the already-mentioned conjugated polymers.

Since the pioneering work on conductive polyacetylene by MacDiarmid, Heeger, and Shirakawa [23], CPs have been mainly investigated for the application in organic electronics—e.g., in organic photovoltaics (OPVs), organic light-emitting diodes (OLEDs), organic field-effect transistors (OFETs), (bio)sensors, or batteries [24,25,26]. The formation of various ROS by conjugated polymers have been known for several years [27]; however, this phenomenon is rather undesirable in the key application of conducting polymers, organic electronics, since it may lead to the oxidative decomposition of the electroactive materials, and thus results in the lowering of the efficiency of the device [28,29,30,31]. Still, what is unfavorable for organic electronic devices appears to be attractive for the biological or photocatalytic applications. Several features make CPs attractive candidates for the generation or detection of singlet oxygen [32]. They are generally characterized by the high absorption in UV–Vis or even near-infrared (NIR) region and an efficient energy transfer [33,34]. Moreover, it has been shown that the energy levels of the S_n_ and T_n_ states, and thus the ISC process [35], can be optimized by increasing the number of conjugated units, which is the basis of so-called “polymerization-enhanced photosensitization” concept [16,36,37]. Conjugated oligomers/polymers can be applied in the photosensitization of ^1^O_2_ either acting as photosensitizer itself or acting as the light-harvesting antennas or backbone to other PS molecules (e.g., dyes, fullerenes) [34,38,39]. In this review paper, the examples of both types of CP–based photosensitizers used in the medical applications, fine chemical synthesis or wastewater treatment–will be discussed.

## 2. Conjugated Polymers as ^1^O_2_ Source in Medical Applications

### 2.1. Photodynamic Therapy (PDT)

Conjugated polymers have received a lot of attention in medical research, due to their good biocompatibility, photostability, relatively easy functionalization, and tunable optical properties. They have been widely studied for the application in imaging (e.g., fluorescence imaging, photoacoustic imaging), regulation of cell activity (e.g., gene expression, enzyme action) and light-activated therapies mainly based on the ROS activity [40,41,42,43]. Reactive oxygen species, including singlet oxygen, commonly occur in biological systems, and they are vital participant in the functioning of the living organisms—e.g., taking part in cell signaling and apoptosis. However, the high concentration of ROS may lead to the oxidative damage of various biomolecules, such as DNA, proteins, which may cause carcinogenesis or age-related diseases. Their origin may be both exo- or endogenous, e.g., they can be produced by UV radiation, but also during the cellular respiration in mitochondria. In the normal cells, the ROS concentration and action are properly balanced, but in cancer cells the level of ROS is significantly higher because of the uncontrolled proliferation, which allows for the imaging of tumor cells. Importantly, cancer cells are typically more sensitive to ROS because of their less effective self-protection [44,45], and hence can be effectively destroyed by ^1^O_2_, etc.

Until now, cancer therapies have mainly included surgery, chemotherapy or radiotherapy. In comparison to them, Photodynamic Therapy (PDT) possesses several advantages, such as higher selectivity, limited interaction with the healthy tissues and consequently reduced number of side effects [10,46]. In PDT, the photosensitizer molecule is introduced into patient’s body and accumulates in the cancer cells. In the next step, PS is activated by the light irradiation with an appropriate wavelength to produce ROS via type I or type II mechanisms. Usually, PDT consists of multiple mechanisms leading to apoptosis or necrosis [34,45,47].

Several factors have to be considered when designing PDT agents. First of all, while PS molecules circulate within the bloodstream, singlet oxygen can also be produced, which would also damage the healthy cells. In order to reduce the side effects and minimize the damage of the normal tissues, the generation of ^1^O_2_ should be precise and controllable. This can be achieved by introduction of pH-, polarity- or wavelength-sensitive systems [48,49]. Since PDT cancer therapy is based on the light-activated process, the penetration of light through tissues has to be considered. The photons in the UV–Vis range are characterized by rather low penetration depths (up to 2.5 mm), while the ones in the near-infrared (NIR) region can penetrate up to 1 cm in tissues. The latter is also reasonably safe to the patient, since it is characterized by minimal phototoxicity [40]. Most of the classical photosensitizers absorb mainly in the range up to 700 nm, like the clinically approved porphyrin-based PDT agents—e.g., photofrins have weak absorbance above 630 nm [50]. Additionally, many organic PSs are strongly hydrophobic, so that their in vivo application in the PDT is limited [51]. Hence, high attention is paid to the new photoactive systems that could overcome the above-mentioned issues.

One of the proposed solutions is the employment of conjugated polymers in PDT therapy. They possess the extended π-conjugation and thus efficiently absorb light in the NIR region, and have high rates of intersystem crossing to triplet states. This, among other things, renders them as promising candidates for the next generation of photosensitizers. The main limitation in application of CPs in biological systems is their highly hydrophobic characters, but this may be overcome by introduction of the polar or ionic side groups that strongly enhance the solubility of such macromolecules in water [52,53,54]. When the cationic or anionic groups are introduced into CP structures, conjugated polyelectrolytes are formed [55]. Conjugated polymers are commonly in the form of nanoparticles in PDT (conjugated polymer nanoparticles (CPNs)) [34,56,57], which can be produced directly during polymerization, under polymerization in micro-/miniemulsions, dispersion polymerization or emulsion polymerization, or after polymerization (postpolymerization strategy), with nanoprecipitation, micro-/miniemulsification or self-assembly processes [58]. Such CPNs usually contain surfactant chains that increase the stability and limit the aggregation in a water solution. Lately, two-photon photodynamic therapy (2PE-PDT) applying conjugated polymers is becoming the promising alternative, thanks to its high spatial resolution and deeper light penetration compared to the classical 1PE-PDT, which may be beneficial for treatment of, e.g., subcutaneous tumors or brain tumors. In this method, the upper excited singlet state of photosensitizers is produced by the absorption of two photons with lower energies. The transition goes via the so-called virtual state. The classical PSs are usually characterized by low two-photon absorption (TPA) cross sections, causing the 2PE process to be ineffective. Therefore, the research is carried out on the application of conjugated polymers (with significantly larger TPA cross section) in 2PE-PDT. Lately, it has been shown that the increase in the conjugation may cause the significant increase in the 2PA cross-section, thus making CPs attractive candidates for the 2PE-PDT strategy [59]. In one-photon and in two-photon PDT approaches, CPs can be applied either in the pristine form (acting as a photosensitizer itself) or in combination with the classical photosensitizers, such as porphyrins [6,60]. In the latter case, the conjugated polymers act as carriers and/or additionally as the light-harvesting antennas that, thanks to the energy transfer to PS, significantly boost the efficiency of singlet oxygen photogeneration. The energy transfer from the π-conjugated backbone to photosensitizer unit may occur via Förster resonance energy transfer (FRET) or bioluminescence energy transfer (BRET) [61].

**Figure 2:**
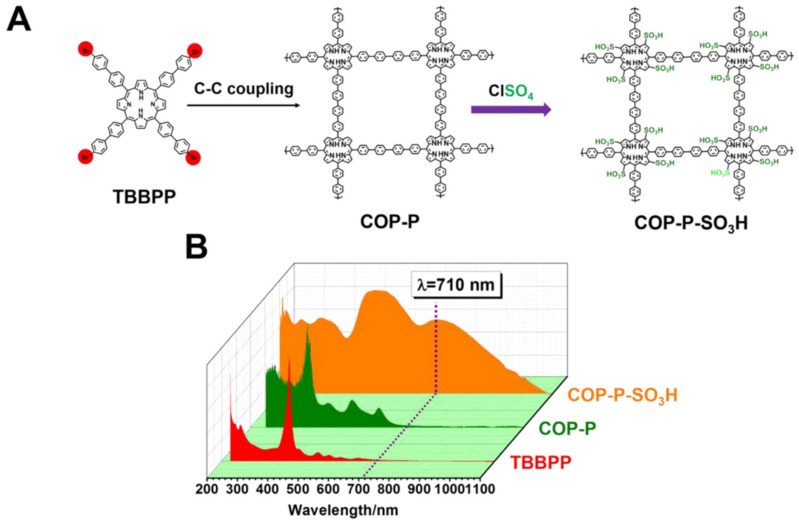
(**A**) Strategy for the formation of the two-dimensional porphyrin-based conjugated polymer for photodynamic therapy; (**B**) UV–Vis spectra of the aqueous solution of substrate and products. Reprinted with permission from [50]. Copyright (2016) American Chemical Society.

The PDT agents consisting of the CPs acting as a PS can be, first of all, obtained by the formation of the two-dimensional system of the covalently bound classical π-conjugated photosensitizers, such as porphyrins. This coupling can be achieved by, e.g., Yamamoto Ullmann cross-coupling, as shown in Figure 2A. The consecutive sulfonation enhances dispersion in water. The resulting CP-based photosensitizer possesses strong absorbance up to 1100 nm, i.e., the region of light that penetrates through tissues, contrary to the monomer that absorbs mainly in the UV–Vis region (Figure 2B) [50].

**Figure 3:**
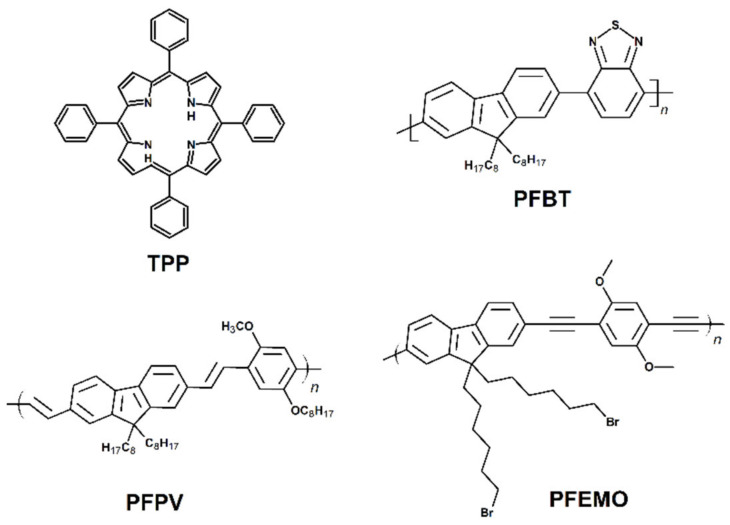
Chemical structure of TPP and fluorene-based polymers (poly[(9,9-dioctylfluorenyl-2,7-diyl)-alt-co-(1,4-benzo-{2,1′,3}-thiadiazole)] (PFBT) and poly[9,9-dibromohexylfluorene-2,7-ylenethylene-alt-1,4-(2,5-dimethoxy) phenylene] (PFEMO) (PFEMO), investigated in [53,62,63], respectively) used to obtained conjugated polymers nanoparticles for photodynamic therapy.

**Figure 4:**
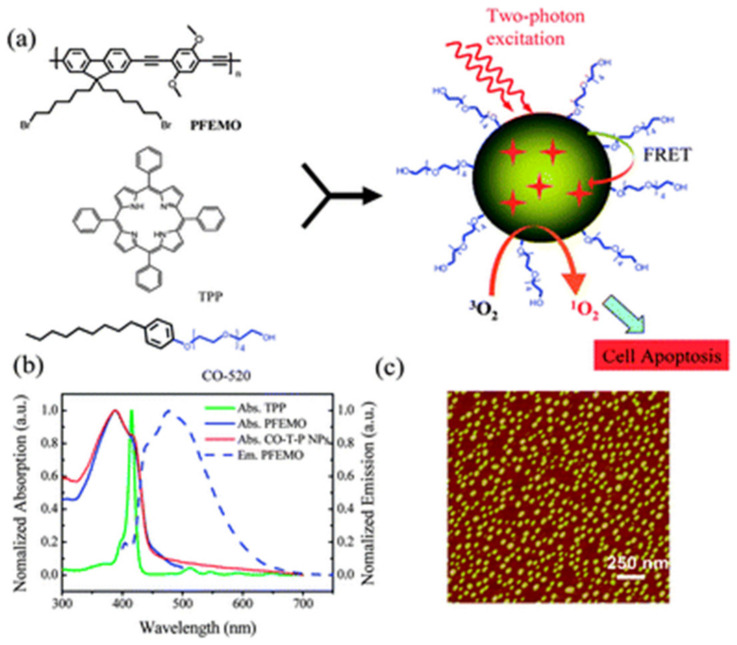
(**a**) Scheme of the formation of conjugated polymer nanoparticles (CPNs) for two-photon photodynamic therapy (two-photon Photodynamic Therapy (2PE-PDT)), (**b**) normalized absorption spectra of TPP, PFEMO and resulting CPNs (solid lines), and emission spectra of PFEMO (dashed line), (**c**) AFM imaging of CPNs on a mica substation. Republished with permission of The Royal Society of Chemistry from [63]; permission conveyed through Copyright Clearance Center Inc.

**Figure 5:**
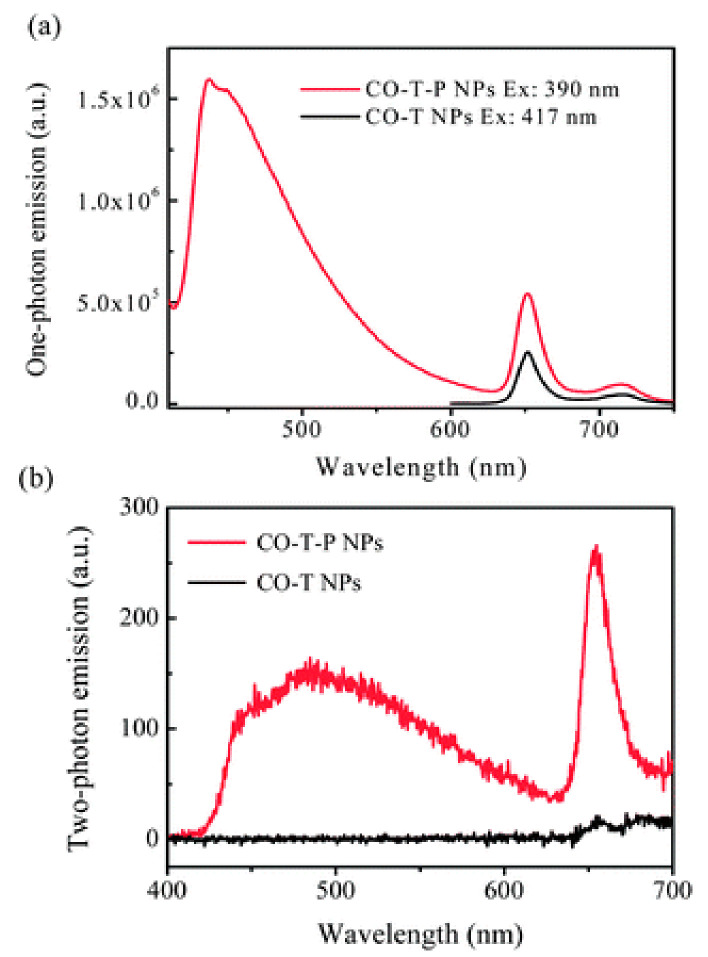
(**a**) Emission spectra of CPNs with or without PFEMO under excitation at their individual absorption maximum (390 nm for CO-T-P NPs and 417 nm for CO-T NPs); (**b**) comparison of emission spectra of CPNs with or without PFEMO under two-photon excitation at 800 nm. Republished with permission of The Royal Society of Chemistry from [63]; permission conveyed through Copyright Clearance Center Inc.

Next to the dye-based CPs, other π-conjugated building blocks have been reported as promising for the light-induced therapy. Let us first consider systems in which CP acts as a carrier and light-harvesting antenna for the classical PSs. The noncovalent incorporation of the PS molecule inside CPNs can be achieved thanks to the hydrophobic interactions or electrostatic interactions, leading to the encapsulation of the PS’s molecules inside the CPs nanoparticles [33]. Various polyfluorene-based polymers, such as poly[(9,9-dioctylfluorenyl-2,7-diyl)-alt-co-(1,4-benzo-{2,1′,3}-thiadiazole)] (PFBT), poly[{9,9-dioctyl-2,7-divinylene-fluorenylene}-alt-co-{2-methoxy-5-(2-ethylhexyloxy)-1,4-phenyl-ene}] (PFPV), poly[9,9-dibromohexylfluorene-2,7-ylenethylene-alt-1,4-(2,5-dimethoxy) phenylene] (PFEMO) (Figure 3) or poly{9,9-bis[6″-(bromohexyl)-fluorene-2,7-ylenevinylene]-co-alt-1,4-(2,5-dicyanophenylene)} (PFVCN), have been reported as supports for the porphyrin photosensitizers, significantly increasing the efficiency of ^1^O_2_ generation under one- or two-photon excitation [53,62,63,64]. In the case of TPP-PFBT system, almost 100% efficient energy transfer was observed and nearly 50% quantum yield of ^1^O_2_ production [53]. A similar quantum efficiency was also reported for the other polyfluorenes. The exemplary system for the 2PE-PDT consisting of TPP photosensitizer and PFEMO as light-harvesting antenna is given in Figure 4. As shown in Figure 4b, the emission spectra of PFEMO nicely overlaps with the absorption bands of TPP photosensitizer. When the one-photon emission spectra of nanoparticles with or without PFEMO are compared (Figure 5a), one can easily see that the intensity of the TPP emission at ca. 650 nm is significantly boosted when polyfluorene is present, which confirms the energy transfer from PFEMO to TPP. Under two-photon excitation (Figure 5b) the nanoparticles without PFEMO show only weak emission, because TPA of porphyrin is relatively low, but it is about 20-times larger in the polyfluorene-containing CPNs [63]. Considering the polyfluorenes as light-harvesting antennas for the 2PE-PDT, it has been shown that the value of TPA can be significantly boosted by introduction of ethynylene or vinylene groups and even more by the incorporation of the electron withdrawing cyano groups [65].

The polyfluorene-vinylene-phenylene-based conjugated polyelectrolyte can act itself as a source of ROS in 2PE-PDT. Similarly to the TPP-containing systems, the presence of the -CN groups attached to the phenyl ring causes a significant increase in the two-photon cross section area and the quantum yield of singlet oxygen photogeneration [66].

Interestingly, when the above-mentioned energy transfer from the light-harvesting chain to PS is incomplete, the double action is possible–the imaging/detection of the cancer cells and the photodynamic action against them, as shown for the polythiophene-porphyrin dyad [54] or iridium (III) complex with covalently attached polyfluorene units. With such π-conjugated systems the imaging guided PDT can be carried out [67], and it can be even more effective when CPNs with additional red-emitting dye components are used [68].

**Figure 6:**
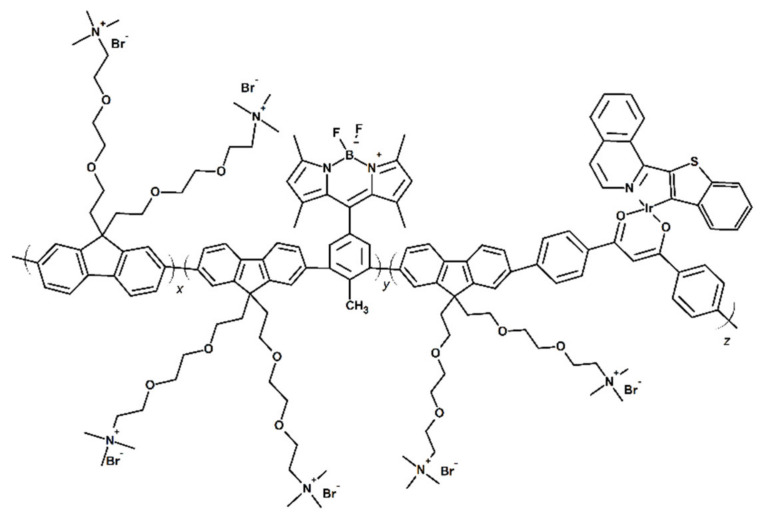
Schematic representation of the polyfluorene-based conjugated polymer with covalently attached Ir (III) complex and BODPIY units (investigated in [49]).

Though, the noncovalent doping of PSs inside CPNs is usually rather straightforward, it may result in the lowered efficiency of ^1^O_2_ photogeneration (because of the aggregation of PS inside nanoparticles) or uncontrolled leakage of PS molecules [51]. Alternatively, the photosensitizer units can be covalently attached to the conjugated polymers either as a pendant group or incorporated in the main chain [33,49,51]. Jiang et al. have recently reported the semiconducting polymer nanoparticles (Figure 6) consisting in the polyfluorene backbone with covalently attached iridium (III) complex and, additionally, boron dipyrromethene (BODIPY) units acting as the energy donors in the FRET process. The application of two luminophores resulted in a very high quantum yield of singlet oxygen photogeneration. Moreover, such CPNs allow for the mapping of the oxygen levels, thanks to the quenching of the phosphorescence of Ir (III) complex [49].

The conjugated polymers can act in a versatile manner in the biological systems. Upon irradiation they can form singlet oxygen for photodynamic therapy [56], but can also show NIR light-induced photothermal action against cancer cells [40,51]. This versatility of CPs is the basis for the development of the complex multifunctional systems for the anticancer therapy. First of all, if properly designed, CPs can act not only as PSs, but also as a platform for carrying other therapeutic agents such as small interfering RNA (siRNA) [69]. Second of all, when the semiconducting polymer nanomaterials are introduced into a patient’s body, the production of ^1^O_2_ molecule (and thus consumption of ^3^O_2_) may also cause the selective cleavage of the linkers within PDT agent, resulting in the release of other therapeutic molecule (e.g., enzyme or drug). This cleavage can be induced either by singlet oxygen, as in the case of –CH_2_-S-C(CH_3_)_2_-S-CH_2_- or –S-CH=CH-S-, or initiated by the local hypoxia. Such remote activation approach, presented by, e.g., Li and Pu, can initiate local chemotherapy resulting in, e.g., DNA damage [40]. It has been reported that self-assembled CPNs consisting of poly(cyclopentadithiophene-alt-benzothiadiazole) grafted with PEG and bromoisophosphoramide mustard intermediates as chemodrugs can present double action when activated by NIR light—i.e., generation of singlet oxygen, and the release of the chemodrug upon hypoxia-cleavage of the linker [40].

In the third case, when Photothermal therapy (PTT) is considered, the additional effects relying on the conversion of light into thermal energy may occur and may result in the ablation and death of the cancer cells. The combination of two types of conjugated polymers into one nanoparticle may give rise to the synergistic therapeutic action of PDT and PTT against tumors. This approach was shown in the work of Feng et al., which describes (1) a fluorene-benzothiadiazole-based polymer and (2) a thiophene-thiadiazolo-quinoxaline-based polymer, acting as PDT and PTT agents, respectively [70]. The resulting CPN showed a double effect against cancer cells, thanks to the significant yield of singlet oxygen photogeneration and high light-to-heat conversion.

**Figure 7:**
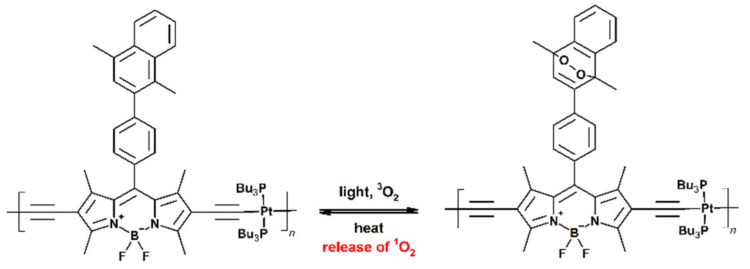
Schematic representation of PDT agent with ^1^O_2_-carrier units (investigated in [71]).

Finally, it has to be remembered that the oxygen level is one of the key factors that has be taken into account in PDT, since its lower value may significantly limit the production of singlet oxygen and thus the efficiency of the whole PDT process. Li et al. proposed an attractive solution for this issue, based on the application of photosensitizers containing dimethylnaphthalene units acting as the singlet oxygen carriers. The ^1^O_2_ molecules are further released in the tumor cells thanks to the NIR-activated photothermal effect (Figure 7) [71].

### 2.2. Photodynamic Antimicrobial Chemotherapy (PACT)

The rapid increase in the number of antibiotic-resistant pathogens has initiated the process of searching for new, nonantibiotic methods of action against microbes. Here, reactive oxygen species, especially singlet oxygen, appear to be promising candidates, since they show high effectiveness against bacteria, viruses and fungi. This antimicrobial character of ROS was the background for the development of photodynamic antimicrobial chemotherapy (PACT). ROS action against microorganisms is nonselective and highly effective. Since they attack microbes in a versatile manner, there is rather small possibility of development of the microbes’ resistance, which is extremely important from an epidemiological point of view [47,72,73]. One of the possible applications of the biocidal properties of ROS is in light-activated antimicrobial coatings, which has received high research interest in recent years [11,74,75]. In this case, it is more favorable to have a photosensitizer to absorb in the visible region so that the solar or conventional indoor light are efficiently used in PACT action.

**Figure 8:**
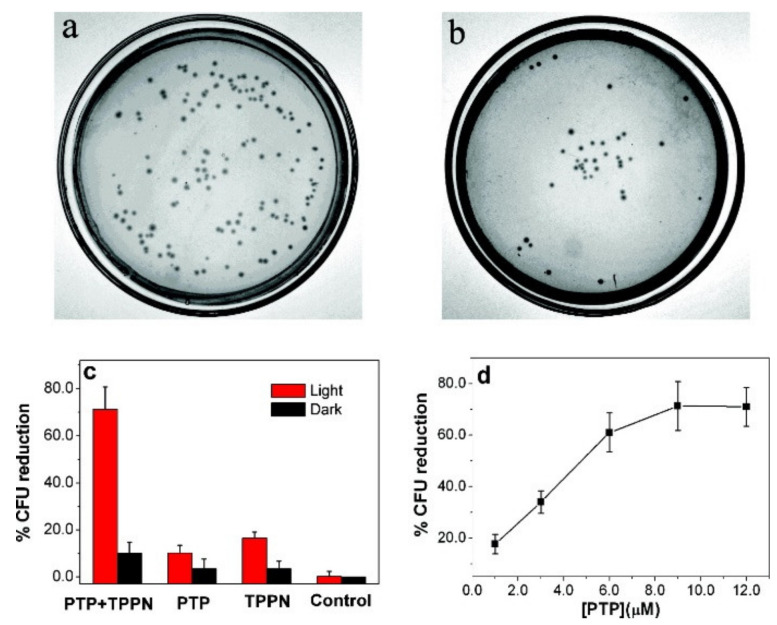
(**a**) Number of colony forming units (cfus) of *Escherichia coli* (*E. coli*) for control without photosensitizer in dark; (**b**) Cfu of *E. coli* suspension incubated with PTP/TPPN and irradiated with white light; (**c**) biocidal activities of PTP/TPPN, PTP, and TPPN toward *E. coli* in the dark and under white-light illumination for 5 min. (**d**) Reduction in cell viability of PTP/TPPN complex toward *E. coli* as a function of PTP concentration. Reprinted with permission from [76]. Copyright (2009) American Chemical Society.

As in the case of PDT therapy, CPs for the PACT application can act as a light-harvesting support for the common photosensitizers, not producing ROS themselves. In the work of Xing et al., the polythiophene–porphyrin system was formed based on the electrostatic interactions between an anionic CP backbone and positively charged porphyrin, and it showed light-activated antimicrobial action (Figure 8a,b). Although the covalent bond between units is not formed, still the energy transfer from the visible light-absorbing polythiophene enhances the production of singlet oxygen in the action against *Escherichia coli* (*E. coli*) under white light irradiation compared to the experiment in which only porphyrin is light-activated (Figure 8c). The efficiency of the biocidal action increases up to certain extent with the increase in porphyrin concentration (Figure 8d) [76].

The possibility of the use of (cationic) conjugated oligomers or polymers in PACT as the main source of ROS has attracted considerable attention in recent years [77,78,79]. The light-induced biocidal action of singlet oxygen and other types of ROS may be further boosted by the presence of the cationic groups in CPs, such as pendant quaternary ammonium units or cationic imidazolium groups in the main chain [80]. Such positively charged parts interact with the negatively charged bacteria membranes, which in turn allows for the more efficient antibacterial response both in dark and under light [81,82,83]. The mechanism of action of the conjugated oligomers/polymers against Gram-positive and Gram-negative bacteria has been widely discussed in the works of Wang et al. [84,85]. The dark- and light-initiated bactericidal actions were investigated with cationic-conjugated oligo- or polyelectrolytes based on phenylene ethynylene. Due to the structural differences of Gram-negative and Gram-positive bacteria, significant differences were observed in the bactericidal mechanism. Moreover, the mechanism also differs between polymeric and oligomeric agents. Considering the dark activity, in the case of the Gram-negative bacteria, the high molecular weight CPs act only on the bacterial cell surface, while oligomers can cause damage also to the cytoplasm. The thicker cell envelope of Gram-positive bacteria is, however, resistant towards penetration of both cationic poly- and oligo-CPs. It has been shown that singlet oxygen and the follow-up ROS cause the damage to the cell wall of the Gram-positive bacteria, while with the Gram-negative one they act on the cell wall and cellular components, such as DNA or proteins.

**Figure 9:**
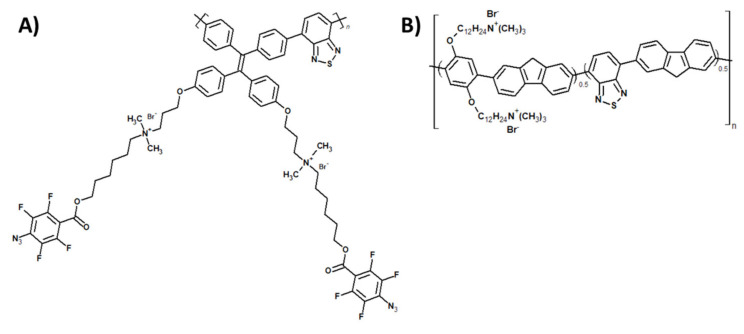
(**A**) Tetraphenylethene- and (**B**) fluorene-co-phenylene-based conjugated polymers with benzothiadiazole units, applied as Photodynamic Antimicrobial Chemotherapy (PACT) agents (investigated in [87,88], respectively).

Among the π-conjugated monomers, benzothiadiazole (BT) is one of the most commonly used in PACT as a source of singlet oxygen in the donor–acceptor systems. For example, it has been shown that molecular doping of the thiophene-based polymer nanoparticles with an electron withdrawing BT group results in the improved photoinactivation of *E. coli* and *Bacillus subtilis* (*B. subtilis*). Importantly, the reported nanoparticles exhibited high stability and reusability [86]. Similar effect of enhancement of antimicrobial properties was observed when benzothiadiazole was introduced into the main chain of tetraphenylethene-containing [87] or fluorene-co-phenylene-based conjugated polymers [88] (Figure 9A, B respectively). The first system showed high effectiveness against *Staphylococcus aureus* (*S. aureus*), while leaving mammalian cells unaffected, while the latter was effective even against ampicillin-resistant *E. coli*.

The possibility of the application of conjugated polymers in the photoinactivation of fungi has also been investigated. Xing et al. reported that the polythiophene–porphyrin system may be effectively used against *Aspergillus niger* [89], while very recently, Jagadesan et al. have shown that water-soluble imidazolium-containing cationic conjugated polymers exhibit high light-activated antifungal properties against Candida albicans [90]. In both cases, the presence of the cationic units allows for the electrostatic interactions of the CPs with the fungal cell wall, as in the case of bacteria, which in turn enhances the diffusion of ROS into the cell membrane. Finally, the latest work of Monge et al. has shown that the phenylene ethynylene-based conjugated oligomers/polymers can be effectively applied against severe acute respiratory syndrome coronavirus 2 (SARS-CoV-2) with the reduction in the virus of up to 5-log within 5 min [91].

The tremendous development in the application of conjugated polymers in photodynamic action against cancer or various microbes has been observed in recent years. However, several unknowns have to be cleared and some issues, such as hydrophobicity, have to be resolved. Additionally, the strategy for the introduction of CP-based drugs into the body and their short-term and long-term safety to patients are critical for opening the doors for the actual application of CPs in light-activated therapies.

## 3. Conjugated Polymers as ^1^O_2_ Sources in Photo-Oxidation Processes

As a strong oxidizing agent, singlet oxygen is considered as a valuable reagent in organic synthesis. To name a few, ^1^O_2_ can be successfully applied in the Diels-Alder reaction with dienes to form endoperoxides, [2 + 2] cycloaddition reaction to olefins to form dioxetanes, synthesis of hydroperoxides from alkenes or phenols or the oxidation of sulfides to sulfoxides and phosphines to phosphine oxides [1,7,8,92,93]. This versatility makes singlet oxygen attractive not only in the synthesis of fine chemicals, but also for solar light-driven wastewater treatment [94]. In both cases, ROS species are considered as economically and environmentally friendly reagents [95,96]. Keeping in mind that the lifetime of singlet oxygen in the solution phase is very short, it has to be photogenerated in situ in the reaction mixture either by homogenous or heterogeneous sensitizers. Typically solid or solid-supported PSs show lower quantum efficiencies of ^1^O_2_ production, but such an approach may be beneficial for the practical use taking into account the product separation, so as the recovery and reuse of the PS. Additionally, in many cases the solid-supported sensitizers showed higher photostability than the counterparts in the solution phase [1,7,8,97,98]. While considering photocatalytic processes employing light-activated conjugated polymers, care has to be taken to properly identify the type of the reactive species (^1^O_2_, e^−^, H_2_O_2_, etc.) being produced by the catalyst. This can be achieved by applying various specific traps and UV–Vis or EPR spectroscopies [99,100,101,102,103].

### 3.1. Synthesis of Fine Chemicals

**Figure 10:**
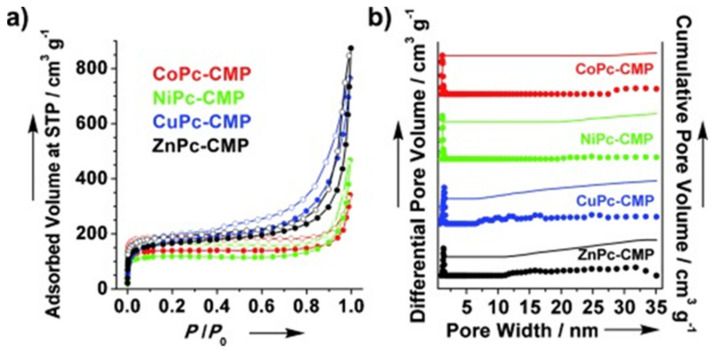
(**a**) Nitrogen sorption isotherms and (**b**) the profiles of the pore size distribution calculated with NLDFT method. Republished with permission of John Wiley & Sons Inc. from [114]; permission conveyed through Copyright Clearance Center Inc.

Thanks to the above-mentioned properties, singlet oxygen has become one of the most widely studied reagents for the synthesis of the fine chemicals, allowing the obtention of various important building blocks of (bio) pharmaceuticals or agrochemicals. In this area, the conjugated microporous polymers (CMPs) have been under high research interest as potential heterogeneous catalysts for more than 10 years now. CMPs are amorphous materials consisting of the π-conjugated monomers covalently linked to form three-dimensional structures [104,105]. They are categorized as a subgroup of porous organic polymers (POPs) [106,107,108]. CMPs are characterized by the strong visible light absorbance resulting from the π-conjugated system and the microporous structure. These properties make them attractive for the visible light-driven photocatalysis either in batch or continuous-flow reactors [109]. The conjugated microporous polymers can consist of the classical photosensitizers, such as porphyrines or phthalocyanines, but structures such as thiophene, perylene or carbazole, commonly studied in the organic electronics, can also serve as CMPs’ building blocks [110,111]. CMPs are obtained in numerous types of coupling or condensation reactions and the chosen synthetic methodology influences optical, redox and morphological properties of the catalyst [112]. From the morphological point of view, several parameters of CMPs have to be well-defined, such as the crystallographic structure characterized by X-ray Diffraction (XRD) or the pore size and the specific surface area that are typically investigated by, e.g., nitrogen sorption measurements (Figure 10). The latter are critical for the heterogeneous catalyst activity and the diffusion of reagents inside the pores and thus they strongly influence the overall efficiency of the photoprocess. Nevertheless, it has been shown that in the case of CMPs, their electronic structure, i.e., proper selection of donor and acceptor units, also plays a vital role in the photoactivity of the polymeric photosensitizers [113].

The CMPs based on phthalocyanines (Pcs) with cobalt, nickel, copper or zinc as the central metal can be synthesized in the polycondensation reaction of amino-substituted Pcs and terephthalaldehyde. The resulting photocatalysts possessed microporous structures and showed broadband absorbance in the Vis–NIR region. Zn- and Cu-containing CMPs showed high activity towards singlet oxygen photogeneration under illumination with 700 nm wavelength [114]. The same group has also shown that various linkers can be used to bind the phthalocyanines that allow for the further functionalization of CMPs structure [115]. Other work on the porphyrin-based CMPs showed their activity in the reaction with 9,10-diphenylanthracene [116], while porphyrin, porphyrin-co-phthalocyanine or subphthalocyanine microporous structures with the broadband absorbance up to 900 nm were reported as an efficient source of ^1^O_2_ in the photo-oxidation of DPBF [117,118,119].

**Figure 11:**
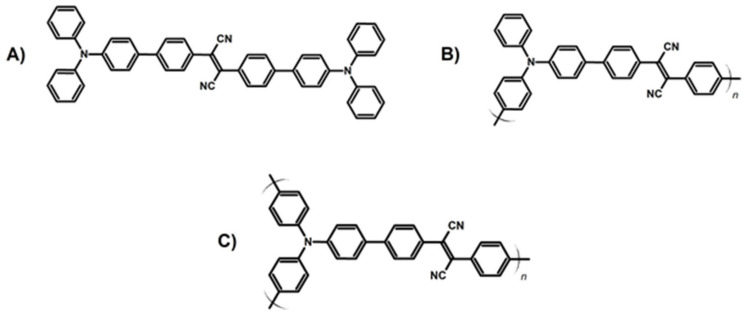
Schematic representation of CMPs based on triphenylamine and fumaronitrile with the increased conjugation lengths from (**A**–**C**) (investigated in [120]).

In the development of the conjugated microporous polymers, donor-acceptor (D-A) systems have gained high interest. Such structures, with tunable photophysical and photochemical properties, can be obtained starting from the D-A dyad monomers [101]. Recently, Wu et al. described the three-step design of conjugated polymeric photosensitizers based on triphenylamine acting as a donor and fumaronitrile serving as an acceptor. The phenyl ring was additionally introduced into π-conjugated monomer structure (Figure 11A) to separate HOMO-LUMO and thus increase the efficiency of the ISC process and ^1^O_2_ production. In the same manner, the corresponding polymer (Figure 11B) possesses higher photogeneration efficiency and broaden absorption. Finally, the crosslinking of the polymeric structure (Figure 11C) caused an increase in the porosity and specific surface area of the catalyst, simplifying separation and reuse. The versatility of the material was shown in the photo-oxidation processes and in the antimicrobial tests [120].

Another nitrogen-containing conjugated monomer, i.e., carbazole, has also shown promising photocatalytic activity for use as a CMP. The introduction of different linker groups, such as benzoselenadiazole or triazines, allows for the tuning of the photophysical and photochemical properties of the resulting catalysts. The reported D-A catalysts have high surface areas and good thermal and chemical stability. They were successfully applied in the light-activated oxidation of α-terpinene to ascaridole, i.e., the anthelmintic drug, or thioanisole to methyl phenyl sulfoxide—i.e., a key intermediate in the synthesis of various pharmaceuticals such as nelfinavir. Moreover, the mentioned CMPs were effectively used in the degradation of mustard-gas simulant [107,121].

The significant photocatalytic and photoredox activities of the benzothiadiazole and benzooxadiazole systems have been demonstrated in the number of visible-light-driven homogenous and heterogonous processes [96,122,123,124,125,126,127]. Zhang et al. have shown that poly (benzothiadiazole)-based CMPs can serve as ^1^O_2_ sources in the oxidation of α-terpinene to ascaridole. The photocatalytic process reached a 90% yield [128]. The introduction of the hydrophilic functional groups in the thiol–yne reaction with 3-mercaptopropionic acid resulted in CMPs easily forming a dispersion in water. Such CMPs were effectively applied in the oxidation of furoic acid under 400 nm irradiation to form 5-hydroxy-2 (5H)-furanone, which is key building block of various biologically active species, such as manoalide, a nonsteroidal anti-inflammatory agent [129].

**Figure 12:**
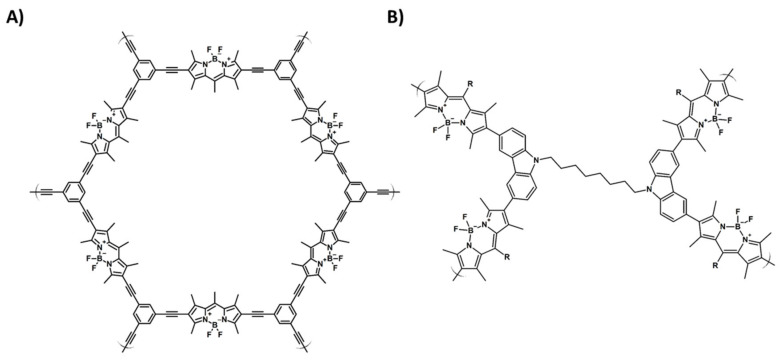
Schematic representation of CMPs based on boron dipyrromethene (BODIPY) with (**A**) 1,3,5-triethynylbenzene or (**B**) carbazole units (investigated in [106] and [135], respectively).

It is well-known that boron dipyrromethene (BODIPY) itself is a very poor photosensitizer; however, it has been shown that the introduction of specific functional groups may lead to an increase in the triplet states probability in BODIPY, hence the increase in its efficiency of ^1^O_2_ photogeneration [130,131,132,133,134]. Liras et al. presented a heavy-atom-free BODIPY-based CMP (Figure 12A) with a microporous structure and high surface area. The material was employed in the light-induced oxidation of thioanisole by singlet oxygen to form methyl phenyl sulfoxide. Compared to the corresponding homogenous catalysts, BODIPY-CMP showed a four-times higher reaction rate and simplified reuse [106]. BODIPY can also be combined with carbazole units (Figure 12B) to form a soluble porous polymeric source of ROS [135]. Tobin et al. also reported the application of the BODIPY-based CMPs in the commercial flow reactor for the photochemical oxidation of α-terpinene with conversion reaching 99% after just 1h [136].

Next to CMPs, another strategy is to use CPs-based photocatalysts in the form of thin layers deposited on the solid support or free-standing nanosheets. In the first case, the layers of conjugated polymers can be formed on the solid support by, e.g., drop-casting, spin-coating or electrochemical polymerization. The glass-supported thin layers of CPs with perylene diimide units obtained by spin-coating were applied in the synthesis of ascaridole from α-terpinene [137]. Moreover, the electrochemical polymerization was proposed as a straightforward process of deposition of the photoactive conjugated films containing phenothiazine and/or fullerene sensitizers [138,139,140,141]. Lately, Zhou et al. reported the polyimide nanosheets formed by combining the perylene core with triazine or heptazine blocks. The resulting conjugated polymer with the optimized π-π stacking showed high photocatalytic activity in the oxidation of amines at room temperature. Both singlet oxygen and superoxide radical anions were identified as the dominant ROS present. The process resulted in the 99% conversion and 100% selectivity after 2h with N-benzyl benzaldimine as the final product [142].

### 3.2. Wastewater Treatment

The high oxidizing properties of singlet oxygen are also useful for the purification of industrial and urban wastewaters [1,94]. Studies in this area mainly focus on the removal of dyes–phenols occurring from, e.g., dye and paper industries—and sulfides, produced in, e.g., the food or petroleum industry, as well as water disinfection from various microorganisms.

**Figure 13:**
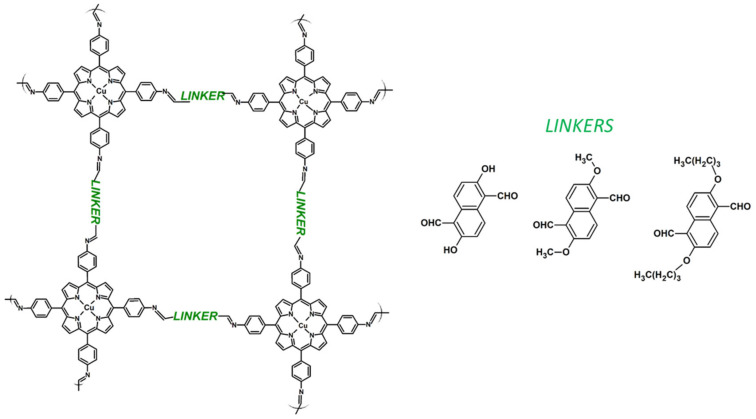
Schematic representation of copper porphyrin-based CMPs with various linkers (investigated in [143]).

Lately, Cui et al. described the CMPs based on the amine-bearing copper porphyrin with various linkers forming imine groups (Figure 13) obtained in microwave-assisted synthesis. The CMPs possessed large surface areas and strong absorptions from 300 up to 1000 nm, and hence were effectively used as a source of ROS (^1^O_2_, OH and O_2_^−^) in the degradation of model dyes from the wastewater. It has also been shown that the best photocatalytic properties were observed for the CMP containing –OH groups, which was assigned to the higher dispersity in water and more advantageous electronic structure, hence indicating that by careful design of the linker CMPs can strongly boost the performance of CMPs [143]. Recently, CMPs have also been effective as a light-activated sources of ROS in the degradation of antibiotics, such as fluoroquinolones [144].

Next to highly porous systems, the 2D- and the linear 1D-conjugated polymers can be also used in light-activated wastewater treatments. The thin layers of electrochemically polymerized phenothiazines showed to be effective in the degradation of the phenol from the aqueous solution under visible light illumination [145], while the dithienoarsole-fluorene-based polymer [146] and 1D polyporphyrin-benzobisoxazole [147] were successfully applied as sources of singlet oxygen in the photodegradation of rhodamine-based dyes. Both CPs were active under visible-light irradiation, could be recycled, and showed high photocatalytic capabilities. Finally, Shen et al. have reported the application of the polyamide-supported benzothiadiazole (BT) photosensitizer in the degradation of bisphenol A and cimetidine and inactivation of cryptosporidium present in water solutions [148].

As shown, the tremendous development in the application of CMPs in photooxidation reactions has been reported in the recent years. The crystallinity, active surface area, chemical purity, and stability of such systems still need to be enhanced to make them competitive to other typically used photocatalysts. This is usually not an issue in the case of the approach based on the thin layers of CPs, but those, on the other hand, usually suffers from the lower overall efficiency of the photooxidation process due to inhibited contact of the reagents with the active surface. Such layers, however, may be advantageous for the application with the continuous-flow photoreactors. Nevertheless, in both forms of CPs more straightforward, environmentally friendly and easy scalable synthetic procedures need to be developed to allow for their industrial use.

## 4. Summary

Based on the presented literature review, it can be stated that the conjugated polymers can be considered as very promising candidates for the next generation of photosensitizers. Thanks to their tunable and advantageous properties, they can meet the requirements to become highly efficient light-activated sources of singlet oxygen for either medical or catalytic purposes. However, a lot still has to be achieved before practical use starts. Above all, the extended (photo)stability of such systems has to be determined, since it has already appeared as a critical issue in the application of CPs in (opto)electronic devices. Nevertheless, as shown by the results of the works published before now, conjugated polymers might be an interesting alternative to classical photosensitizers and thus they deserve high attention and further investigation.
